# Effects of Secondhand Exposure to Heated Tobacco Products on Human Milk Composition

**DOI:** 10.3390/toxics14070563

**Published:** 2026-06-27

**Authors:** Masako Tateno, Katsumi Mizuno, Midori Date, Miori Tanaka

**Affiliations:** 1 Department of Pediatrics, Showa Medical University School of Medicine, 1-5-8 Hatanodai, Shinagawa-ku, Tokyo 142-8666, Japan; masako.t@med.showa-u.ac.jp; 2 The Nippon Foundation Human Milk Bank, 17-10 Nihonbashi-koamicho, Chuo-ku, Tokyo 103-0016, Japan; midori.date@milkbank.or.jp (M.D.); miori.tanaka@milkbank.or.jp (M.T.)

**Keywords:** breastfeeding, cotinine, human milk, secondhand smoke, heated tobacco products

## Abstract

Background: Secondhand exposure to heated tobacco products (HTPs) is increasingly common, but its impact on human milk composition is unclear. We investigated whether secondhand exposure to HTP aerosols affects major human milk components and cotinine concentrations in lactating women. Methods: This observational study included 15 lactating women whose household members used HTPs (secondhand HTP exposure group) and 33 lactating women who did not live with any smokers (non-exposed group). Human milk was analyzed for macronutrients, total solids, energy, lactoferrin, secretory immunoglobulin A (sIgA), calcium, inorganic phosphorus, zinc, and cotinine. Cotinine was measured in all women in the secondhand HTP exposure group and in three women in the non-exposed group. Results: Background characteristics did not differ significantly between groups. No significant differences were observed in lipid, protein, total solids, energy, true protein, lactoferrin, calcium, inorganic phosphorus, or zinc. Carbohydrate concentration differed significantly between the non-exposed and secondhand HTP exposure groups (non-exposed vs. secondhand HTP exposure: 8.20 vs. 8.10 g/dL, *p* = 0.032), although the absolute difference was small. sIgA tended to be higher in the secondhand HTP exposure group (non-exposed vs. secondhand HTP exposure: 1244 vs. 1706 μg/mL, *p* = 0.072). Cotinine concentrations did not differ significantly between groups; qualitative cotinine tests were negative in all samples. Conclusions: Secondhand exposure to HTPs was not associated with clear differences in major human milk components or cotinine concentrations. However, the findings should be interpreted cautiously because of the small sample size and limited cotinine assessment. Larger studies with objective exposure assessment and infant follow-up are needed.

## 1. Introduction

Cigarette smoke contains more than 4000 chemical compounds, including nicotine, and over 60 known carcinogens [1]. Exposure to secondhand tobacco smoke has been shown to adversely affect the health and development of fetuses and infants [2,3,4]. Nevertheless, many women continue to be exposed to secondhand smoke through their partners or living environments. In Japan, around 40% of pregnant women are exposed to secondhand smoke in some form, with reported exposure frequencies of 23.7% for 1–3 days per week and 15.8% for 4–7 days per week in indoor environments such as the home, workplace, and other enclosed settings [5].

In recent years, the use of heated tobacco products (HTPs) has increased, driven by the widespread perception that they are less harmful to health than conventional cigarettes [6,7]. HTPs are devices in which tobacco leaves or reconstituted tobacco products are inserted into a dedicated holder and electrically heated to generate an inhalable aerosol. According to the National Health and Nutrition Survey in Japan (2019), 20–30% of current smokers use HTPs. The nicotine content of HTP aerosols has been reported to be comparable to, or slightly lower than, that of conventional cigarettes [8,9]. Although HTP aerosols contain multiple toxic and harmful chemicals at concentrations considered lower than those in conventional cigarette smoke, several studies suggest that the health risks associated with HTPs may be comparable to those of conventional cigarettes [9,10].

Breastfeeding plays a fundamental role in the healthy growth and development of infants. Studies have shown that when lactating women are exposed to tobacco smoke, nicotine and other chemicals, including carcinogens, pass into human milk [11,12]. Nicotine has been shown to suppress prolactin secretion and the milk ejection reflex, thereby reducing milk production and shortening the duration of breastfeeding [13]. The altered taste of human milk due to nicotine exposure may cause some infants to refuse feeding [13]. Furthermore, maternal smoking during lactation has been reported to alter the composition of human milk, including reduced lipid, energy, and protein concentrations, as well as decreased antioxidant capacity and iodine content [14,15]. Even when mothers themselves do not smoke, exposure to secondhand smoke from smokers in the household has been associated with reduced lipid and protein levels in human milk [11,16].

However, the impact of secondhand exposure to HTP aerosols on human milk composition remains unclear. In this study, we investigated the effects of secondhand exposure to HTPs on human milk composition and additionally measured cotinine concentrations in human milk, as cotinine is the major metabolite of nicotine.

## 2. Materials and Methods

Participants were recruited using a study poster to invite eligible lactating women to participate. The secondhand exposure group consisted of 15 lactating women whose household members used HTPs. The non-exposed group consisted of 33 lactating women who did not live with any smokers; this group included 30 human milk bank donors whose donated milk to The Nippon Foundation Human Milk Bank (TNFHMB) had been consented for research use and 3 lactating women recruited via the study poster for the present study. This study was approved by the Showa Medical University Research Ethics Review Board (approval number: 22-141-B). Informed consent was obtained from all subjects involved in the study.

Secondhand exposure to HTP aerosols was assessed using a questionnaire completed by the participating lactating women. The questionnaire included information on the presence of household HTP users, the number of HTP sticks used per day by household members, and the location of HTP use, including whether HTPs were used in the same room as the mother, in another indoor room, or outdoors/on a balcony. Participants were classified into the secondhand exposure group if they lived with household members who used HTPs. Participants were classified into the non-exposed group if they did not live with any smokers or HTP users.

Human milk samples were collected from participants recruited for the present study and from donated milk provided to TNFHMB with consent for research use. Participants collected human milk samples in human milk storage bags. No specific instructions were provided regarding foremilk or hindmilk collection, and the method of milk expression, such as manual expression or use of a breast pump, was not specified. After collection, human milk samples were stored at −30 °C until analysis. Before analysis, frozen samples were shipped to TNFHMB and thawed in a refrigerator.

The concentrations of lipid, protein, carbohydrate, total solids, energy, and true protein in human milk were measured using a Miris Human Milk Analyzer (Miris AB, Uppsala, Sweden), which uses infrared spectroscopy to quantify milk macronutrients and energy content. Daily quality control (including calibration checks and cleaning) was performed prior to sample analysis using the Miris Calibration Control Kit, Miris Check, and Miris Cleaner (Miris AB). Samples were warmed to 40 °C, sonicated, and then analyzed. As demonstrated in previous studies, this analyzer provides acceptable agreement with reference methods for the measurement of human milk macronutrients [17,18,19].

The concentrations of lactoferrin (LF), calcium (Ca), inorganic phosphorus (IP), and zinc (Zn) in human milk were measured as previously described [20,21]. LF levels were determined by a latex agglutination assay using Latex Test BL Lactoferrin (Biolinks, Takayama, Japan) after 100-fold dilution with the supplied diluent and were measured using a CA-270 Clinical Chemistry Analyzer (Furuno Electric, Hyogo, Japan) [20]. Ca, IP, and Zn were determined by colorimetric assays using Accuras Auto Ca II, Accuras Auto IP, and Accuras Auto Zn (Shino-Test, Tokyo, Japan), respectively. Milk samples were diluted 5-fold with the supplied diluent and measured using the CA-270 Clinical Chemistry Analyzer [21].

Secretory immunoglobulin A (sIgA) was measured by an enzyme-linked immunosorbent assay (ELISA). Samples were centrifuged to separate the fat layer, and the lower aqueous phase was collected for analysis. The concentrations of sIgA were measured using a Secretory Immunoglobulin A ELISA Kit (Immunodiagnostik, Bensheim, Germany).

For cotinine measurement, cotton swabs were immersed in expressed human milk collected in human milk storage bags. The cotton swabs were stored frozen and sent to Kanematsu Wellness Co. (Tokyo, Japan), where cotinine concentrations were measured by ELISA. Donated milk samples from TNFHMB were not used for cotinine measurement because these samples were provided for milk composition analysis and were not collected using the cotton-swab protocol required for cotinine assessment. Therefore, cotinine concentrations were measured only in participants recruited specifically for the present study, including 15 women in the secondhand exposure group and 3 women in the non-exposed group. For qualitative cotinine testing, the cutoff value for positivity was 10 ng/mL.

Statistical analyses were performed using JMP Pro version 17 (SAS Institute Inc., Cary, NC, USA). Group comparisons were performed using the Mann–Whitney U test, with the significance level set at *p* < 0.05. All data are presented as medians.

## 3. Results

In the secondhand HTP exposure group, the most common daily number of HTP sticks smoked by household members was 10–19 (41%), followed by ≥20 (33%) and ≤10 (26%). The main locations for smoking were outdoors (e.g., on a balcony) in 46% of cases, in the same room as the mother in 27%, and in a different room within the home in 27%. Background characteristics of the non-exposed and secondhand exposure groups are presented in Table 1. There were no significant differences between the non-exposed and secondhand HTP exposure groups in gestational age at delivery (38.0 vs. 39.0 weeks, *p* = 0.24), postpartum week at the time of milk expression (17.0 vs. 16.0 weeks, *p* = 0.76), maternal age (34.0 vs. 31.0 years, *p* = 0.19), birth weight (3085 vs. 3074 g, *p* = 0.93), or parity (2.0 vs. 1.0, *p* = 0.17).

Human milk composition is shown in Table 2. There were no significant differences between the non-exposed and secondhand HTP exposure groups in median concentrations of lipid (3.20 vs. 3.90 g/dL, *p* = 0.48), protein (1.10 vs. 1.00 g/dL, *p* = 0.41), total solids (12.8 vs. 13.1%, *p* = 0.44), energy (68.0 vs. 72.0 kcal/dL, *p* = 0.39), or true protein (0.90 vs. 0.80 g/dL, *p* = 0.97). Carbohydrate concentration differed significantly between the non-exposed and secondhand. HTP exposure groups (8.20 vs. 8.10 g/dL, *p* = 0.032).

There were no significant differences between the two groups in lactoferrin (1349 vs. 1377 μg/mL, *p* = 0.53), calcium (30.4 vs. 28.0 mg/dL, *p* = 0.63), inorganic phosphorus (5.35 vs. 5.20 mg/dL, *p* = 0.74), or zinc (128.0 vs. 129.0 μg/dL, *p* = 0.69). Although the difference did not reach statistical significance, sIgA tended to be higher in the secondhand exposure group (1244 vs. 1706 μg/mL, *p* = 0.072).

With respect to cotinine concentrations, there was no significant difference between the non-exposed group and secondhand HTP exposure group (3.1 vs. 3.4 ng/mL, *p* = 0.37). Within the secondhand HTP exposure group, cotinine concentrations did not differ significantly between women whose household members smoked in the same room and those whose household members smoked in a different area (3.4 vs. 3.3 ng/mL, *p* = 0.54). Qualitative cotinine tests were negative in all samples.

## 4. Discussion

In this pilot study, we examined the impact of secondhand exposure to HTPs on human milk composition and cotinine concentrations. Although a statistically significant difference in carbohydrate concentration was observed between the secondhand exposure and non-exposed groups, carbohydrate concentration was only slightly lower in the secondhand HTP exposure group by 0.10 g/dL (non-exposed vs. secondhand exposure: 8.20 vs. 8.10 g/dL; *p* = 0.032). This finding should be interpreted with caution given the number of comparisons performed, as it may reflect a type I error. Lactose, the main carbohydrate in human milk, contributes to the osmotic pressure of milk and serves as a major energy source for infants [22]. However, the small difference observed in this study is expected to have only a limited impact on infant growth and development.

The immune-related component sIgA tended to be higher in the secondhand HTP exposure group, although this difference did not reach statistical significance (*p* = 0.079). sIgA is a major immune factor in human milk that contributes to mucosal defense and can limit bacterial translocation through the infant gut mucosa [23]. Previous studies have reported that sIgA concentrations in the milk of smoking mothers were approximately 27% lower than those in non-smokers, although the difference did not reach statistical significance [24]. Conversely, other reports have found higher sIgA concentrations in the milk of mothers with a history of smoking [25], suggesting that the association between smoking-related exposure and milk sIgA concentrations may not be uniform. Because immune-related milk components can be influenced by maternal physiological and inflammatory conditions, and smoking-related exposure has been linked to oxidative stress and reduced antioxidant capacity [26], these factors may contribute to variability in milk sIgA. Larger studies with more detailed exposure assessment will be needed to determine whether secondhand exposure to HTP aerosols is associated with meaningful changes in immune-related components of human milk.

Research investigating the effects of secondhand exposure to aerosols from HTPs or electronic cigarettes on human milk composition is extremely limited. In animal experiments, Al-Sawalha et al. exposed lactating rats to e-cigarette aerosols for 1 h daily from postnatal day 4 through day 20 [27]. Maternal exposure during lactation was associated with reduced milk fat content and lower maternal serum leptin levels compared with controls, whereas milk protein and lactose concentrations did not differ markedly. Offspring nursed by exposed dams also exhibited metabolic and endocrine alterations, including higher blood glucose and lower insulin levels. These findings suggest that exposure to e-cigarette aerosols during lactation may influence not only milk lipid content but also metabolic and hormonal profiles in both mothers and offspring.

In humans, secondhand exposure to electronic cigarettes has been described in a report by Ballbè et al. [28]. In that report, cotinine was detected in the milk of a mother living with an electronic cigarette user, suggesting that nicotine can, in practice, be transferred to infants via human milk as a result of secondhand exposure.

In contrast to these reports, our study did not detect significant differences in lipid concentrations, other major human milk components, or cotinine concentrations. Several factors may account for these discrepancies, including differences in exposure intensity, environmental conditions, and product characteristics. In our study, most household members in the secondhand exposure group smoked outdoors, and exposure in the same room as the mother occurred in less than 30% of cases, whereas the experiment by Al-Sawalha et al. involved forced exposure in an enclosed space. In addition, all qualitative cotinine tests were negative in the secondhand exposure group, suggesting that the overall level of exposure was relatively low. Although the interval between the most recent secondhand exposure and milk collection was not recorded, cotinine has a relatively long half-life (approximately 6–30 h) [29]. In addition, cotinine concentrations in human milk exhibit less diurnal variation than nicotine and remain relatively stable over a 24 h period [29]. Therefore, the lack of precise information on the timing of the last exposure is unlikely to fully explain the absence of between-group differences in cotinine in the present study. Moreover, the studies by Al-Sawalha et al. [27] and Ballbè et al. [28] focused on electronic cigarettes, whereas our study examined HTPs, which differ from electronic cigarettes in heating mechanisms and aerosol constituents. These differences in exposure conditions and product characteristics may partly explain the inconsistent findings among studies.

Importantly, the absence of statistically significant differences in the present study should not be interpreted as evidence that secondhand exposure to HTP aerosols during lactation is harmless. Rather, the present findings should be considered hypothesis-generating and warrant confirmation in larger studies incorporating objective exposure assessment, quantitative biomarker measurements, and longitudinal infant follow-up.

## 5. Limitations

This study has several limitations. First, the sample size was relatively small (*n* = 48), which may have limited the statistical power to detect modest differences between groups and increased the risk of type II error. This limitation is particularly important for the cotinine analysis. Although the non-exposed group consisted of 33 lactating women for the analysis of human milk composition, donated milk samples from TNFHMB were not used for cotinine measurement because they were not collected using the cotton-swab protocol required for cotinine assessment. Therefore, cotinine concentrations were measured in only three non-exposed participants. This limited number is insufficient for robust statistical comparison, and the cotinine findings should therefore be interpreted with caution.

Second, secondhand exposure was assessed solely based on self-reported information regarding household members’ use of HTPs. Although information on the number of HTP sticks used per day and the location of HTP use was collected, objective exposure assessment was not performed. Specifically, we did not measure environmental nicotine concentrations, airborne particulate matter, room ventilation conditions, or biomarkers of exposure in maternal blood or urine. In addition, precise information regarding the frequency, duration, and timing of secondhand exposure before milk collection was not available. Therefore, misclassification of exposure status cannot be excluded. Some women classified as non-exposed may have experienced low-level environmental exposure outside the home, whereas women in the secondhand exposure group may have had minimal effective exposure if HTP use occurred primarily outdoors. The absence of objective and quantitative exposure assessment limited our ability to evaluate dose–response relationships and may have attenuated potential differences between groups.

Third, detailed information on HTP characteristics and patterns of use, such as product brand, heating temperature, puffing frequency, and duration of use, was not collected. Because HTPs differ in heating mechanisms and aerosol constituents, individual differences in product type and usage behavior may have influenced the actual exposure level.

Fourth, the milk collection protocol was not fully standardized. No specific instructions were provided regarding foremilk or hindmilk collection, and the method of milk expression, such as manual expression or use of a breast pump, was not specified. Because human milk composition can vary according to sampling conditions, this may have introduced variability in the measured milk components.

Finally, this was an observational study and therefore cannot establish a causal relationship between secondhand exposure to HTP aerosols and changes in human milk composition. In addition, longitudinal follow-up of infant health outcomes was not performed. Therefore, the present findings cannot determine whether secondhand exposure to HTP aerosols affects infant growth, metabolism, or long-term health, and should not be interpreted as evidence that such exposure is harmless during lactation.

## 6. Conclusions

In this study, we examined the effects of secondhand exposure to HTPs on major human milk components and cotinine concentrations. We did not observe significant differences in protein, lipid, or other major milk components between the secondhand exposure and non-exposed groups. However, these findings should be interpreted with caution because of the small sample size, the limited number of non-exposed participants included in the cotinine analysis, and the lack of objective exposure assessment.

Given the limited evidence regarding secondhand exposure to HTP aerosols during lactation, the absence of statistically significant differences in this study should not be interpreted as evidence that HTP exposure is harmless. Household members should be encouraged to avoid using HTPs around lactating women and infants. At the same time, because breastfeeding provides substantial nutritional and immunological benefits, decisions regarding breastfeeding should be made by carefully considering both the benefits of breastfeeding and the potential risks of secondhand exposure. Further studies with larger sample sizes, objective assessment of exposure, and long-term follow-up of maternal and infant outcomes are needed to clarify the effects of secondhand exposure to HTPs on human milk composition and child health.

## Figures and Tables

**Table 1:** Characteristics of the study participants.

	Non-Exposed Group *N* = 33	Secondhand Exposure Group *N* = 15	*p*-Value
Gestational age at delivery (weeks)	38.0 (38.0–40.0)	39.0 (38.0–40.0)	0.24
Postpartum week at milk expression (weeks)	17.0 (12.0–24.0)	16.0 (9.5–28.0)	0.76
Maternal age at milk expression (ages)	34.0 (31.0–36.0)	31.0 (29.0–34.5)	0.19
Birth weight (g)	3085 (2630–3310)	3074 (2861–3136)	0.93
Parity	2.0 (1.0–3.0)	1.0 (1.0–2.0)	0.17

Data are expressed as the median (interquartile range).

**Table 2:** Human milk composition in the non-exposed and secondhand exposure groups.

	Non-Exposed Group (*n* = 33)	Secondhand Exposure Group (*n* = 15)	*p*-Value
Lipid (g/dL)	3.20 (2.30–4.30)	3.90 (2.65–5.35)	0.48
Protein (g/dL)	1.10 (0.90–1.20)	1.00 (1.00–1.25)	0.41
Carbohydrate (g/dL)	8.20 (8.10–8.30)	8.10 (8.00–8.15)	0.032
Total solids (%)	12.8 (11.6–13.5)	13.1 (12.4–14.5)	0.44
Energy (kcal/dL)	68.0 (57.0–75.0)	72.0 (63.0–85.5)	0.39
True protein (g/dL)	0.90 (0.80–1.00)	0.80 (0.80–1.00)	0.97
Lactoferrin (μg/mL)	1349 (1095–1578)	1377 (1101–1896)	0.53
sIgA (μg/mL)	1244 (1099–1591)	1706 (1272–2043)	0.072
Ca (mg/dL)	30.4 (26.0–32.7)	28.0 (26.5–32.4)	0.63
IP (mg/dL)	5.35 (4.90–5.55)	5.20 (4.53–5.98)	0.74
Zn (μg/dL)	128.0 (74.0–181.0)	129.0 (80.5–222.0)	0.69

Data are expressed as the median (interquartile range).

## Data Availability

The raw data supporting the conclusions of this article will be made available by the authors on request.

## Abbreviations

The following abbreviations are used in this manuscript:

HTPs	Heated tobacco products
TNFHMB	The Nippon Foundation Human Milk Bank
sIgA	Secretory immunoglobulin A
ELISA	Enzyme-linked immunosorbent assay
